# NMR-Based Milk Metabolomics

**DOI:** 10.3390/metabo3020204

**Published:** 2013-04-02

**Authors:** Ulrik K. Sundekilde, Lotte B. Larsen, Hanne C. Bertram

**Affiliations:** 1 Department of Food Science, Aarhus University, Kirstinebjergvej 10, Årslev DK-5792, Denmark; E-Mail: hannec.bertram@agrsci.dk; 2 Department of Food Science, Aarhus University, Blichers Allé 20, Tjele DK-8830, Denmark; E-Mail: lottebach.larsen@agrsci.dk

**Keywords:** milk, NMR spectroscopy, metabolomics, metabolites

## Abstract

Milk is a key component in infant nutrition worldwide and, in the Western parts of the world, also in adult nutrition. Milk of bovine origin is both consumed fresh and processed into a variety of dairy products including cheese, fermented milk products, and infant formula. The nutritional quality and processing capabilities of bovine milk is closely associated to milk composition. Metabolomics is ideal in the study of the low-molecular-weight compounds in milk, and this review focuses on the recent nuclear magnetic resonance (NMR)-based metabolomics trends in milk research, including applications linking the milk metabolite profiling with nutritional aspects, and applications which aim to link the milk metabolite profile to various technological qualities of milk. The metabolite profiling studies encompass the identification of novel metabolites, which potentially can be used as biomarkers or as bioactive compounds. Furthermore, metabolomics applications elucidating how the differential regulated genes affects milk composition are also reported. This review will highlight the recent advances in NMR-based metabolomics on milk, as well as give a brief summary of when NMR spectroscopy can be useful for gaining a better understanding of how milk composition is linked to nutritional or quality traits.

## 1. Introduction

Milk of human and bovine origin is important to the diet respectively during infancy and adulthood. Lipids and lactose are the two major caloric nutrients in milk. Furthermore, milk also contains a wide variety of bioactive compounds, including immunoglobulins and other immune proteins, peptides, nucleotides, oligosaccharides, and metabolites. The composition of milk is influenced by a range of different factors, e.g. diet [1], genetics [2], number and stage of lactation [3], and, for bovine milk, also seasonal variation [4], somatic cell count [5], and milk processing [6]. These factors may have remarkable quantitative effects on milk nutrients, as well as on the physical and technological properties of milk ((e.g., coagulation properties, heat stability, and fermentation quality of the milk). The coagulation properties are influenced by a range of different factors such as casein micelle size [7], protein composition [8], genetic variants of proteins [9], udder health [10], milk pH, and the availability of minerals [11,12]. Therefore, the knowledge about the chemical composition of milk is important for our understanding of its nutritional value, including bioactive compounds, its technological properties, and the potential use of biomarkers in milk as a diagnostic tool.

Milk metabolites can originate from multiple cell types or metabolisms in the organism, and the different origin and sources of metabolites contribute to the variability of milk metabolite profiles. Metabolites in milk often reflect metabolic activity in the mammary gland. In the mammary gland, several pathways and mechanisms are in place to transport compounds across the cell membrane [13]. Furthermore, a paracellular pathway is open in special cases, such as during pregnancy, involution, and in inflammatory states such as mastitis [13]. Variation in the flux via the pathways, and especially the paracellular pathway can change the milk metabolite profile markedly. The paracellular route will change with mastitis and will also reflect milk yield, with low-yielding animals having proportionally more paracellular leakage [14]. Moreover, metabolites may also be secreted by microorganisms present in raw milk [15,16], from somatic cells, or they may originate from enzymatic reactions occurring in the milk [17].

Metabolomics is the high-throughput identification and quantification of metabolites representing the metabolome [18]. Metabolomics has been extensively applied in the areas of pharmaceutical sciences [19] and in food and nutrition sciences [20]. A comprehensive review of the techniques and the use of metabolomics in food science is available [20]. Metabolomics studies often include a high number of samples and there is thus a requirement of high-throughput analytical methods. In general, there are three analytical techniques that dominate the metabolomics methodologies; high-resolution proton nuclear magnetic resonance (NMR) spectroscopy (^1^H NMR), liquid chromatography mass spectrometry (LC-MS), and gas chromatography mass spectrometry (GC-MS). Each of the different methodologies has its advantages and disadvantages, and often the techniques complement each other. In metabolomic studies, ^1^H NMR is an attractive method, as it is by nature a non-targeted approach, requires minimal sample preparation, and detects all mobile hydrogen-containing molecules. Compared with LC-MS and GC-MS, one of the advantages of NMR spectroscopy is the direct and quantitative relationship between molar concentration and the intensity of the NMR resonances. Moreover, it is a non-destructive technique; consequently, the sample can be analyzed in multiple consecutive NMR experiments, or additionally be analyzed by other analytical techniques after the NMR experiments are performed. A disadvantage is the fact that an NMR spectrometer is expensive. However, the running costs are lower compared with other techniques, partly due to the minimal sample preparation requirement. Another disadvantage of NMR spectroscopy is the sensitivity and sample size requirement, whereas the MS techniques excel. However, in the case of milk metabolomic applications, difficulties associated with sample size are rarely a problem. For a more thorough comparison between analytical techniques refer to [20]. The compounds and metabolites identified by high-field ^1^H-NMR spectroscopy of biofluids and foodstuffs commonly include sugars, small organic acids, vitamins, nucleotides, and aromatic compounds.

In this review, we will highlight recent advances in high-resolution ^1^H NMR-based milk metabolomics applications and summarize important analytical aspects of milk metabolite profiling. Knowledge of milk metabolite profiles can be of utmost importance in order to study both bovine or human physiology and milk-processing capabilities at the dairies. Moreover, the present review will briefly discuss applications of NMR spectroscopy in investigating properties of milk fat and protein fractions. Collectively, this knowledge can be used as a basis for understanding how milk’s nutritional and technological quality can be optimized.

## 2. Milk Metabolomics

Various types of NMR experiments have been used in the study of milk. The studies include the analysis of milk fat composition [21], characterization of structural changes in caseins [22,23], casein micelles [24,25,26], whey proteins [27,28,29], proteins and phosphorylated compounds in milk [30,31,32], low-field NMR relaxation studies (for a review see [33]). More recently, ^1^H high-resolution NMR spectroscopy has also been applied as a metabolomics approach on the study of milk [34,35,36,37,38].

**Figure 1:**
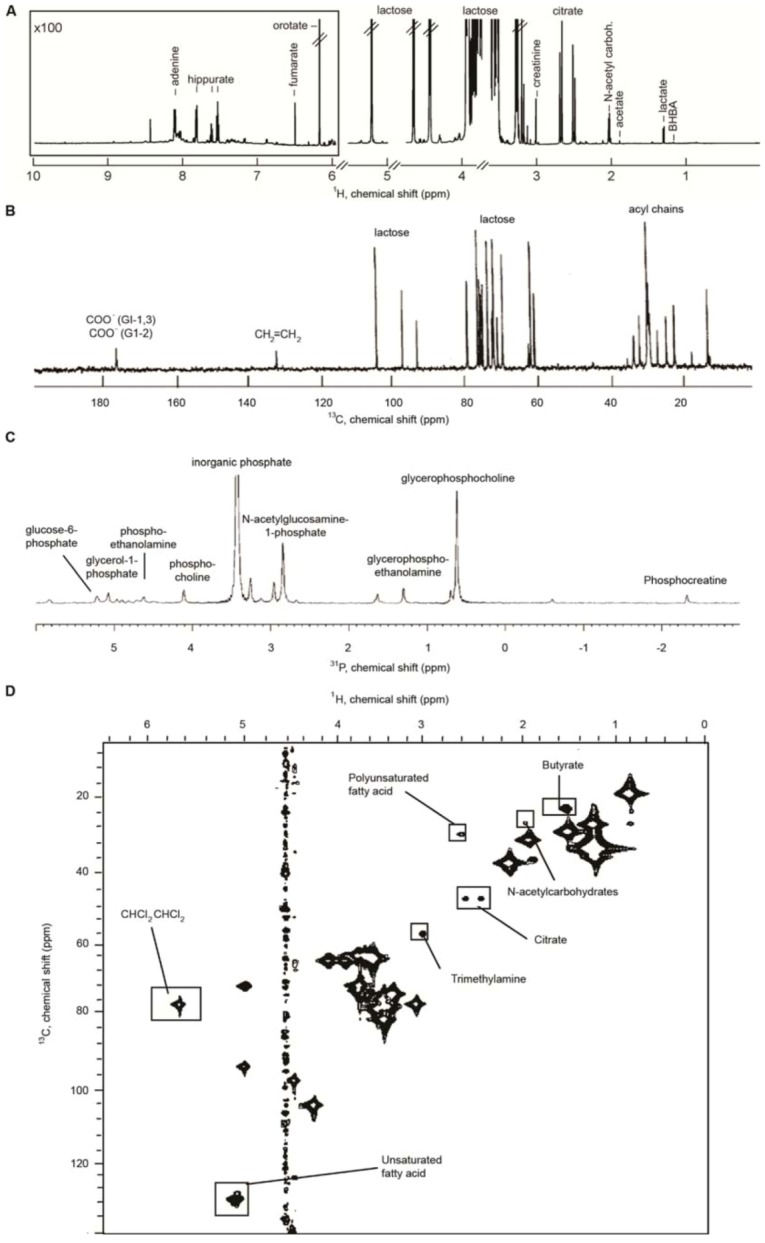
(**A**) ^1^H NMR spectrum of cow milk ultrafiltrate [Adapted from 37]. (**B**) ^13^C NMR spectrum of cow milk [Adapted from 39]. (**C**) ^31^P NMR spectrum of cow milk ultrafiltrate [Adapted from 40]. (**D**) ^1^H-^13^C HSQC NMR spectrum of cow milk [Adapted from 41]. Reprinted with permission [37,39,40,41].

High resolution 1D ^1^H, ^13^C, and ^31^P NMR spectra, as well as a 2D ^1^H-^13^C HSQC NMR spectra of milk or milk ultrafiltrate, are shown in Figure 1. The figure shows many of the metabolites present in milk, and it highlights which features are most abundant in milk. The ^1^H NMR spectrum of milk ultrafiltrate is dominated by resonances from lactose, but a number of other metabolites are also visible (Figure 1A). The ^13^C NMR spectrum of whole milk is also dominated by lactose and, additionally, resonances originating from the acyl chains in milk lipids (Figure 1B). In the ^31^P NMR spectrum of milk ultrafiltrate inorganic phosphate is the most abundant phosphorous species in milk, but more than 10 other phosphorous-containing molecules are also present (Figure 1C). The 2D NMR spectra of a heteronuclear ^1^H-^13^C HSQC experiment on whole milk shows resonances from mainly lactose and lipids (Figure 1D).

### 2.1. Detection of Milk Metabolites and Biomarkers by High-Resolution Proton NMR Spectroscopy

One of the first attempts using high-resolution ^1^H NMR spectroscopy to characterize the complete milk metabolome was carried out using commercially available whole milk [39]. Hu et al. [39] performed both 1D and 2D NMR spectroscopy and assigned a number of metabolites, including citrate, creatinine, N-acetylcarbohydrates, and phospholipids. Furthermore, Hu et al. [41] attempted to quantitate lipids, lactose, citrate, N-acetylcarbohydrates, butyrate, and trimethylamine by ^1^H-^13^C 2D heteronuclear single quantum coherence (HSQC) NMR spectroscopy [41]. Cross peak intensities in 2D NMR experiments are affected by several experimental factors such as relaxation times, mixing time, evolution time, and uneven excitation profiles, and thus the quantitative aspect of the applied 2D HSQC may be questioned. However, the studies by Hu and colleagues proved that NMR spectroscopy was able to identify a considerable number of metabolites, thus providing the basis for metabolomics-based studies. However, the colloidal nature of milk and restricted mobility of the different milk components have impact on the ^1^H NMR analysis as both factors lead to a lower-quality NMR spectrum. The casein micelles and the fat globules, holding the majority of the protein and fat molecules in milk, are large aggregates with low mobility compared to smaller components, e.g. lactose, choline, carnitine and citrate, which are mainly found as free moving (i.e. non-micellar) components in the whey [42]. Consequently, the presence of large molecules and aggregates in milk may result in broad, interfering signals in the ^1^H NMR spectrum. This is especially important in heat-treated milk as aggregation occurs between β-lactoglobulin and κ-casein. Thus, sample preparation presents a crucial step in obtaining high-quality data for NMR-based metabolomics from both raw and heat-treated milk samples.

In a study of the biochemical variability of milk from individual cows in early and late lactation, 25 metabolites were identified by ^1^H NMR spectroscopy [35], and the same approach was used to assess the metabolic status of the cow based on potential biomarkers [36]. In these two studies, the individual raw milk samples were skimmed and passed through a filter with a 10 kDa cut-off threshold in order to remove lipids and proteins [35,36]. Klein *et al.* found that β-hydroxybutyrate (BHBA), phosphocholine, and glycerophosphocholine could be used as biomarkers for ketosis [35], and especially the ratio between glycerophosphocholine and phosphocholine was identified as showing great promise in the diagnosis of ketosis [36]. Ketone bodies in milk are known to be biomarkers for ketosis [43].

**Table 1:** List of chemical shift values and proton assignments for milk metabolites identified by ^1^H NMR spectroscopy reported in the literature. Reprinted with permission [35].

Metabolite	Assignment	^1^H Chemical Shift (ppm)	Reference
Acetate	CH_3_	1.92	[35]
Acetone	CH_3_	2.24	[35]
cis-aconitate	CH_2_	3.15	[44]
Adenine	CH-8	8.12	[37]
Adenine	CH-2	8.13	[37]
Alanine	CH	3.79	[35]
Alanine	CH_3_	1.48	[35]
β-hydroxybutyrate	CH_3_	1.20	[35]
Betaine	3 × CH_3_	3.26	[35]
Butyrate	CH_3_	0.90	[37]
Carnitine	3 × CH_3_	3.21	[35]
Carnitine	CH_2_	2.44	[35]
Carnitine	N-CH_2_	3.43	[35]
Carnitine	CH	4.57	[35]
Choline	3 × CH_3_	3.18	[35]
Choline	O-CH_2_	4.06	[35]
Choline	N-CH_2_	3.51	[35]
Citrate	CH_2_	2.52	[39]
Citrate	CH_2_	2.72	[39]
Creatine	CH_3_	3.79	[39]
Creatine	CH_2_	2.88	[39]
Creatinine	CH_2_	4.06	[35]
Creatinine	CH_3_	3.05	[35]
Ethanolamine	O-CH_2_	3.83	[35]
Ethanolamine	N-CH_2_	3.15	[35]
Formate	CH	8.45	[45]
Fucose	CH_3_	1.25	[44]
Fumarate	CH	6.52	[37]
Galactose α	CH	4.07	[35]
Galactose α	CH	3.81	[35]
Galactose β	CH	4.57	[35]
Galactose β	CH	3.49	[35]
Galactose-1-phosphate	CH-1	5.38	[44]
Glucose	CH_2_	5.1	[44]
Glucose-1-phosphate	CH-1	5.51	[44]
Glutamate	γ-CH_2_	2.36	[44]
Glycerophosphocholine	O-CH_2_	4.32	[35]
Glycerophosphocholine	N-CH_2_	3.65	[35]
Glycine	CH_2_	3.57	[35]
Hippurate	CH_2_-2,6	7.84	[37]
Hippurate	CH-4	7.64	[37]
Hippurate	CH_2_-3,5	7.54	[37]
Isobutyrate	CH_3_	1.16	[44]
Isoleucine	δ-CH_3_	0.93	[37]
Lactate	CH_3_	1.32	[35]
Lactate	CH	4.11	[35]
Lactose (total)	CH-1’	4.45	[35,39]
Lactose (total)	CH-5’	3.73	[35,39]
Lactose (total)	CH-2	3.94	[35,39]
Lactose (total)	CH-2’	3.54	[35,39]
Lactose (total)	CH_2_-6	3.67	[35,39]
Lactose (total)	CH_2_-6’	3.78	[35,39]
Lactose α	CH-1	5.23	[35,39]
Lactose α	CH_2_-6	3.88	[35,39]
Lactose α	CH-3	3.59	[35,39]
Lactose α	CH-4'	3.96	[35,39]
Lactose α	CH-4	3.66	[35,39]
Lactose α	CH-5'	3.84	[35,39]
Lactose β	CH-1	4.67	[35,39]
Lactose β	CH-4	3.66	[35,39]
Lactose β	CH-2	3.29	[35,39]
Lactose β	½ CH_2_-6	3.96	[35,39]
Lactose β	CH-3	3.6	[35,39]
Lactose β	½ CH_2_-6	3.81	[35,39]
Lactose β	CH-5	3.84	[35,39]
Lecithin	3 × CH_3_	3.12	[39]
Lecithin	CH_2_-4	4.22	[39]
Lecithin	CH_2_-3	4.18	[39]
Lecithin	CH_2_-5	3.75	[39]
Lecithin	CH_2_-5	3.83	[39]
Malonic acid	CH_2_	3.11	[44]
3-Methylhistidine	α-CH	3.97	[35]
3-Methylhistidine	β-CH	3.30 / 2.25	[35]
3-Methylhistidine	CH_3_	3.74	[35]
3-Methylhistidine	δ-CH	7.14	[35]
3-Methylhistidine	ε-CH	8.09	[35]
Methionine	γ-CH_2_, S-CH_3_	2.15	[44]
*N*-acetylcarbohydrates	CH_3_	2.06	[39]
Ornithine	γ-CH_2_	1.80	[44]
Orotate	CH	6.20	[37]
Phosphocholine	O-CH_2_	4.16	[35]
Phosphocholine	N-CH_2_	3.58	[35]
Phosphocholine	3*CH_3_	3.18	[35]
Phosphocreatine	CH_3_	3.03	[35]
Phosphocreatine	CH_2_	3.93	[35]
Taurine	S-CH_2_	3.43	[35]
Taurine	N-CH_2_	3.27	[35]
Triethylamine-N-oxide	CH_3_	3.27	[35]
Urea	NH_2_	5.79	[44]
Valine	CH_3_	1.05	[44]

A more costly disease to the farmers and dairies is mastitis, which involves inflammation of the udder. Mastitis inflammation can either be subclinical or clinical, and in both cases milk somatic cell count (SCC) is elevated and the milk quality is affected. Several milk metabolites are known to be biomarkers of elevated SCC or mastitis, including acetate [46], lactose [47], and lactate [46]. Recently, we identified an association between novel milk metabolites and moderately increased SCC in milk from individual dairy cows [37]. BHBA, butyrate, and isoleucine were found to be present in higher concentrations in milk with elevated SCC, whereas fumarate and hippurate were found to be present in lower concentrations in milk with elevated SCC [37]. The observed milk metabolites reported in the literature along with their respective ^1^H chemical shift and proton assignment are listed in Table 1. Thus, the use of ^1^H NMR spectroscopy for milk profiling to detect biomarkers can help to diagnose diseases such as subclinical and acute ketosis or mastitis or be used in routine screening as milk is readily available, resulting in optimal herd disease management.

Milk metabolomics can also be used in the investigation of physiology of lactation, as the milk metabolites reflect the metabolic activity of the mammary glands. Recently, a study investigated the relationship between milk and blood serum metabolites in dairy cows [48]. The study by Maher *et al.* also revealed a correlation between trimethylamine levels in serum and milk and an inverse correlation between serum valine and milk fumarate. Moreover, the study identified that the concentration of milk protein could be associated with low levels of orotate, citrate, and lactose in the milk [48].

### 2.2. Metabolomics-Assisted Elucidation of Important Technological Milk Quality Parameters

The technological properties of milk are important for the dairy industry, as they influence the processing capabilities of milk to high quality products, and thus, the technological properties are also important for the consumer. Important milk processing steps at the dairies are rennet coagulation and syneresis of curd, which is associated with cheese-making. Rennet coagulation is an enzymatic hydrolysis of κ-casein producing para-κ-casein and hydrophilic caseinomacropeptide (CMP) or glycomacropeptide (GMP), depending on the glycosylation status of the individual κ-casein molecules. This first step in the rennet coagulation process is followed by aggregation and curd formation due to a reduction in colloidal stability of the micelles caused by the removal of the hydrophilic macromolecules [42].

Many factors are known to affect milk coagulation including species, breed, protein composition, and genetic variants of proteins [9]. Until recently, the relationship between the milk metabolites and the coagulation properties of the milk was largely unexplored. Results from a metabolomic study on bovine milk have identified associations of variations in milk metabolite profiles with coagulation properties [34]. We identified that choline, carnitine, citrate, and lactose could be used in prediction of milk coagulation properties [34]. Choline was found to be positively associated, while carnitine, lactose, and citrate were found to be negatively associated with well coagulation parameters [34]. However, presently it remains unclear precisely how these metabolites affect the milk coagulation process, but one hypothesis could be associated with posttranslational modifications of the κ-caseins. Posttranslational modifications of caseins are known to affect the coagulation ability of milk [49,50]. The positively charged choline may affect the phosphorylation sites of the κ-casein variants, whereby the degree of phosphorylation is lowered or choline may shield or bind to the negative charges of the phosphate group that is added by the phosphorylation.

Another technological parameter is milk stability, as the milk is processed into various dairy products. Heat treatment, including ultrahigh temperature (UHT) treatment is known to induce chemical changes in the milk [51]. ^31^P NMR spectroscopy has been used to investigate the stability of phosphorus-containing compounds in UHT-treated milk during storage [52]. The authors of that study also analyzed changes in the milk metabolome after inoculation with *Pseudomonas* and identified several metabolites that could potentially be used as indicators of spoilage and thus used in quality control. Moreover, ^31^P NMR spectroscopy has also been shown to be able to determine the casein content following pasteurization or UHT treatment [31]. Multiple phosphorus-containing compounds have been identified in a study of buffalo milk by ^31^P NMR spectroscopy, including metabolites in energy metabolism (galactose-1-phosphate, glycerol-1-phosphate, and glucose-6-phosphate, and *N*-acetylglucosamine-1-phosphate) [40]. Moreover, the study identified that cow and buffalo milk contained the same classes of phosphorylated compounds with some variability between the two species [40].

### 2.3. Milk Authentication and Control of Geographical Origin

The commercial value of many dairy products is closely associated with the origin, species, and composition of the milk used. Cows, buffalo, goat, sheep, and additional animal species all deliver milk which is processed into various dairy products of different qualities and commercial values. Examples of traceability of geographical origin include NMR spectroscopic studies on cow and buffalo milk [45,53]. Also ^1^H HR-MAS NMR applications have been useful in quality control and traceability of Italian buffalo mozzarella [54]. Additionally, ^1^H NMR spectroscopy has been used to identify milk mixtures of skimmed milk samples of cow’s milk and sheep’s milk [55]. Moreover, NMR spectroscopy has been used to screen for compounds in milk and infant formula that have impact on health, and a study revealed that NMR spectroscopy could identify milk adulteration with melamine [56], which was used to mimic an increase in protein concentration in China [57]. Thus, NMR spectroscopy is a highly useful technique for the quality control and adulteration control of milk. Accordingly, NMR spectroscopy has been found to be suitable for routine non-targeted food analysis in quality control which can increase food safety significantly.

### 2.4. Metabolomics and Nutritional Quality of Milk

^1^H NMR spectroscopy has also been used as a tool to validate the labeling in relation to milk nutrition [58]. This specifically impacts consumers suffering from lactose intolerance due to lactase non-persistence [59]. The use of NMR spectroscopy to quantify lactose concentration in milk samples has several advantages compared with existing technologies for estimation of lactose concentration with increased sensitivity as the most prominent advantage [58]. The nutritional quality of milk is also an important parameter due to milk being an essential part of the diet of both infants and adults worldwide. In an earlier study, the choline content of human breast milk in the first three weeks after birth was compared with bovine milk and infant formula by use of ^1^H NMR spectroscopy [60]. The observed choline species included free choline, phosphocholine, glycerophosphocholine, phosphatidylcholine, and sphingomyelin. Holmes *et al.* identified that total choline content in human colostrum at birth is lower than in mature milk seven days post-partum, which correlates well with the acceleration in growth that the neonate experiences at this time point [60]. Additionally, the authors of this study speculate that for pre-term infants, the choline content available in human milk is not sufficient for the rapid growth these infants experience, as the metabolic activity is higher in pre-term infants compared with full-term infants [60]. Pre-term human milk and formula have recently been investigated [38], but the number of metabolites identified was low compared with studies performed on bovine milk [35,44]. Marincola *et al.* identified differences between human breast milk and infant formulae and studied differences between milk from women who had delivered early and late pre-term, and suggested differences in milk metabolite profiles. However, the study included relatively few available samples, and it was therefore not possible to conclude which metabolites differed in milk from women who delivered early or late pre-term, respectively [38]. However, the use of NMR spectroscopy metabolomic studies of both pre-term and full-term human milk is showing great potential, especially if the number of metabolites identified can be increased.

Another recent milk metabolomic study was performed on human milk and rhesus macaque monkey milk [61]. The study identified several differences between the milk metabolome of human and rhesus milk; amino acids and oligosaccharides were generally more abundant in human milk samples, whereas other metabolites including glycerophosphocholine, hippurate, and trimethylamine-N-oxide were more abundant in rhesus milk samples [61]. Additionally, the study assessed the overall differences in serum and urine metabolomes, as well as established the milk microbiome using 16S rRNA gene sequencing in order to investigate the use of rhesus monkeys as a model for human nutrition [61].

The composition of phospholipids in milk is also of interest in relation to nutrition. Phospholipids participate in many biological roles and are key components of cell membranes. Phospholipids are the backbone of plasma membranes and form the unique double plasma membrane encompassing the milk fat globules (milk fat globule membrane (MFGM)). Investigations of the plasma membrane constituents may offer the possibility to identify indirect relationships between milk metabolites and phospholipids and phosphoproteins. Although some of the phospholipids also are detectable by ^1^H-NMR spectroscopy, the utilization of ^31^P-NMR spectroscopy offers increased resolution in case of phospholipids and it may therefore be more suitable to detect the phosphorus-containing compounds using ^31^P-NMR spectroscopy. Twelve phospholipid species and total phospholipid and lipid content of camel, mare, cow and human milk were profiled and quantified in a recent ^31^P-NMR study [62]. The study identified significant differences in the phospholipid profiles between breeds [62], which can be useful to the dairy industry as these phospholipids can be purified and concentrated and then added to infant formula or other dairy products as functional ingredients.

Nutritional studies of milk based on NMR metabolomics can aid in establishing the optimal composition of infant formula and milk directed towards pre-term, full-term, and older babies, as well as adults.

### 2.5. Impact of Genes on Milk Metabolite Variability

As previously mentioned, the origin of milk metabolites is normally the mammary epithelial cells producing the milk components. Milk metabolites can be divided into groups; metabolites present in milk for the benefit of the receiver (neonate), metabolites resulting from metabolic activity in the mammary gland, or metabolites which passed through the paracellular pathway from the blood or interstitial fluid. Some of these metabolites can be altered through feeding/diet [63]. Recently, we have studied the influence of genetics on milk metabolite profiles [44]. NMR spectroscopy was used to provide the metabolite profiles of milk from 371 Holstein–Friesian cows, which were also genotyped using the bovine HD single nucleotide polymorphism (SNP) chip. A genomic relationship matrix was then calculated based on the SNP data, and used as a random factor in a model with herd and lactation-stage as fixed factors. In total, 31 metabolites were identified and relatively quantified, and for each individual metabolite, the heritability and breeding value was estimated using the model [44]. The identified metabolite heritabilities were in the range from 0 (lactate) to 0.87 (BHBA) [44], with a value of 1 indicating a purely genetic explanation for variability.

Moreover, results from the genome-wide association study (GWAS) revealed genome-wide significant QTL for malonate (BTA2 and BTA7), galactose-1-phosphate (BTA2), cis-aconitate (BTA11), urea (BTA12), carnitine (BTA25), and glycerophosphocholine (BTA25) [44]. Moreover, 21 chromosome-wide significant QTL were also identified for other milk metabolites. Thus, these data suggest that genomic selection in order to change bovine milk metabolite composition is possible, which could have a profound effect on milk technological properties or milk nutritional quality.

### 2.6. Summary

To summarize, NMR-based milk metabolomics enable a systematic study of the association of milk metabolites to various important milk characteristics. There are several technological properties of high economic importance to the farmers and dairies which can be analyzed and quantified using NMR spectroscopy of bovine milk. Moreover, the milk metabolite profiling and investigations of genetic impact on metabolites can help unravel the physiology of lactation, which still have some blind spots. Additionally, NMR-based milk metabolomics is a useful tool in routine analysis used both in quality control and as screening for diseases. The different studies of milk metabolites using NMR-based metabolomics is listed in Table 2.

**Table 2:** A summary of milk NMR-based metabolites and metabolomic studies reported in the literature. BHBA, β-hydroxybutyrate; GC-MS, gas chromatography mass spectrometry.

Factor under investigation	Metabolite(s)	Analytical technique	Reference(s)
Coagulation properties	Choline, carnitine, citrate, lactose	^1^H NMR	[34]
Somatic cell count	BHBA, lactate, lactose, hippurate, acetate, fumarate, butyrate	^1^H NMR	[47]
Metabolic status of cows	Acetone, BHBA	^1^H NMR & GC-MS	[35]
Quality control	Citrate, N-acetylcarbohydrates, trimethylamine, lecithin	^1^H NMR	[39,41]
Quality control	Lactose	^1^H NMR	[58]
Classification of milk blends from different species	*N*-acetyllactosamine, citrate, acetamide, phosphocreatine	^1^H NMR	[55]
Milk authenticity and assessment of adulteration	Melamine	^1^H NMR & GC-MS	[56]
Milk authenticity	Glycerol 1-phosphate, glucose 6-phosphate, phospholipids	^31^P NMR	[40]
Genetic influence on milk metabolites	BHBA, orotate, carnitine, malonate	^1^H NMR	[44]
Infant formula, pre-term, and full-term human milk	Lactose, maltose	^1^H NMR	[38]
Nutrition	Phospholipids	^31^P NMR	[62]
Spoilage, storage	Phosphoglycerides	^31^P NMR	[52]
Nutrition, human milk	Choline, phosphocholines	^1^H NMR	[60]
Human and rhesus macaque milk, nutrition	Amino acids, oligosaccharides, glycerophosphocholine, hippurate	^1^H NMR	[61]
Associations of blood-milk metabolites	Trimethylamine, lactose, citrate, dimethylsulphone, orotate, fumarate, valine	^1^H NMR	[48]

## 3. Experimental Considerations

### 3.1. NMR Spectroscopy

In order to apply NMR spectroscopy of milk and dairy products, there are several considerations that must be taken into account. They include a large variance in size and mobility of the different components, which may lead to differences in widths of resonance lines and relaxation times. Additionally, small changes in chemical shift of metabolites can occur due to small differences in pH or by intermolecular interactions. Moreover, the high sample complexity may lead to significant overlapping or masking of resonances from multiple components. Altogether, these issues will lead to loss of information obtained from NMR spectroscopy, and in milk, this may lead to masking of resonances from minor components with a similar chemical shift as major components, e.g. lactose. There are several ways to circumvent these issues and include removal of specific milk components or fractions, *i.e.* centrifugation to remove the lipids (skimming), precipitation of proteins like ultracentrifugation to sediment casein micelles or acid precipitation of the caseins, both resulting in milk serum or whey fractions, ultrafiltration, or extractions using different solvents. A number of NMR experiments may also be used including total correlation spectroscopy (TOCSY), two-dimensional J-resolved spectroscopy (J-RES), diffusion ordered spectroscopy (DOSY), Carr-Purcell-Meiboom-Gill sequence (CPMG), and a number of 2D homonuclear and multinuclear NMR experiments can also be used [64]. Some metabolites are sensitive to fluctuations in pH or osmolarity, e.g. citrate. Consequently, the associated resonances with these may shift up- or down-field. The citrate resonances at δ 2.73 ppm and δ 2.53 ppm are known to be sensitive to changes in pH and osmolarity [65]. Identification of metabolites is performed by comparison with existing knowledge from the literature [35,39,41,44], the Human Metabolome Database [66], Biological Magnetic Resonance Data Bank [67], spike-in experiments, and 2D NMR spectroscopy.

### 3.2. Data Handling

Following NMR acquisition, each spectrum is multiplied by a line-broadening function during the Fourier Transformation and the resulting spectra are often processed manually by baseline and phase corrections. Subsequently, a number of preprocessing steps must be performed in order to obtain reliable data suitable for multivariate data analysis, including alignment, data reduction (binning), normalization, and scaling.

The chemical shift changes due to intermolecular interactions or pH changes across highly complex biological samples can be both locally or for the entire chemical shift range. Consequently, these changes must be adjusted in order to compare samples. This can be performed using alignment [68,69] or binning [70]. Binning can be achieved by integration of a fixed width, typically 0.04 ppm [70], or by variable width of chemical shifts [71,72,73,74]. The disadvantages of using binning includes a loss in resolution and thus the possibility that NMR resonances from different molecules might be placed into the same bin, thereby both complicating interpretation and obscuring important biological variation. Alignment may also introduce artifacts into the aligned NMR spectra depending on the algorithm used. Furthermore, it is imperative that it is identical resonances across samples that are being aligned. Sample dilution or NMR instrument settings may influence the NMR data and, thus, normalization is another crucial preprocessing step prior to data analysis. Data can be normalized by different methods, but the most common methods include normalization to sample volume, to TSP, to an internal metabolite known to be stable across the sample set, to total intensity, and probabilistic quotient normalization [75]. Normalization to total intensity may not always be applicable in cases where the NMR spectrum is dominated by resonances from a single metabolite. Thus, in the case of milk, normalization to total intensity will in effect normalize to lactose concentrations. Scaling of NMR data is also an important parameter to consider in order to allow influence from low-abundant metabolites in the multivariate models. Normally, Pareto scaling is favored over scaling to unit variance for scaling of NMR data [76]. Pareto scaling is the division with square root of the standard deviation, whereas unit variance scaling is the division with the standard deviation.

### 3.3. Data Analysis

NMR-based metabolomic datasets consist of a large number of samples and variables. Thus, advanced analytical tools suited for data visualization and overview of large datasets are essential. Multivariate data analysis (MVDA) is therefore applied to examine systematic variation in a data matrix in order to identify underlying variables that contribute to differences between samples. Briefly, MVDA techniques include principal component analysis (PCA), partial least squares discriminant analysis (PLS-DA), hierarchical cluster analysis (HCA), and Random Forest (RF) (Table 3). PCA is used to provide a linear transformation of the original variables measured in samples into a substantially reduced set of uncorrelated variables, the principal components (PC) [77]. Thus, PCA is an unsupervised method and is consequently used as an exploratory tool to identify the main variation in the dataset and to identify how samples are related or differ from each other. Supervised analytical tools are also important in identifying variables that describe differences between known classes of samples. PLS-DA can be used to enhance the separation of samples into classes by rotating PCA components. The most important features of both unsupervised and supervised MVDA techniques are summarized in Table 3, along with some of their associated advantages and disadvantages.

**Table 3:** A summary of commonly applied multivariate data analysis approaches in metabolomics studies. Abbreviations: PCA, principal component analysis; PLS-DA, partial least squares discriminant analysis; OPLS-DA, orthogonal partial least squares discriminant analysis; HCA, hierarchical cluster analysis; RF, Random Forest.

Technique	Unsupervised / Supervised	Characteristics
PCA	Unsupervised	Exploratory clustering technique extremely useful in identification of differences between observations including variable differences and covariances
PLS-DA	Supervised	Maximum separation between groups of observations is achieved using rotating PCA components. Useful for obtaining information about which variables are involved in class separation
OPLS-DA	Supervised	Systematic variation that is not correlated with classes is removed, which may improve interpretation but not predictivity
HCA	Unsupervised	Exploratory tool to visualize groupings of observations and represented as a tree or dendrogram showing observation homology
RF	Supervised or unsupervised	A learning algorithm which uses an ensemble of decision trees to assign class relationships to observations

## 4. Conclusions

Milk metabolomics represents a very promising field of study and it has the potential to impact primary producers, industry, and consumers. Moreover, studies involving identification of milk metabolomes are important for obtaining a better understanding of mammary gland physiology, food, dairy, and animal science through mammary gland metabolic activity, milk composition, and milk quality. Evidence has been obtained that the milk metabolites detected by NMR-based metabolomics are of importance in relation to milk nutritional quality, technological properties, quality control, and bioactivity. Moreover, milk metabolomics may be used as a diagnostic tool, as biomarkers in other biofluids such as urine or blood plasma are often used for diagnostic purposes, and the finding of biomarkers in bovine milk leads us to propose the possible use of milk as a diagnostic tool, as well.
